# Delta neutrophil index as an early marker of disease severity in critically ill patients with sepsis

**DOI:** 10.1186/1471-2334-11-299

**Published:** 2011-11-01

**Authors:** Byung Hoon Park, Young Ae Kang, Moo Suk Park, Won Jai Jung, Su Hwan Lee, Sang Kook Lee, Song Yee Kim, Se Kyu Kim, Joon Chang, Ji Ye Jung, Young Sam Kim

**Affiliations:** 1 Pulmonary and Critical Care Division, Department of Internal Medicine, Severance Hospital, Seoul, Korea; 2 The Institute of Chest Diseases, Yonsei University College of Medicine, Seoul, Korea

## Abstract

**Background:**

The immature granulocyte count has been reported to be a marker of infection and sepsis. The difference in leukocyte subfractions (delta neutrophil index, DNI) in ADVIA 2120 reflects the fraction of circulating immature granulocytes in the blood. This study evaluated the clinical utility of DNI as a severity and prediction marker in critically ill patients with sepsis.

**Methods:**

One hundred and three patients admitted to the medical intensive care unit with sepsis were studied. DNI (the difference in leukocyte subfractions identified by myeloperoxidase and nuclear lobularity channels) was determined using a specific blood cell analyzer.

**Results:**

Forty four patients (42.7%) were diagnosed with severe sepsis/septic shock. Overt disseminated intravascular coagulation (DIC) occurred in 40 (38.8%). DNI was significantly higher in patients with severe sepsis/septic shock and overt DIC than in patients without (*p *< 0.05). DNI correlated with DIC score (r = 0.54, *p *< 0.001). We observed a monotonic increase in the proportion of overt DIC and severe sepsis/septic shock associated with increasing quartiles of DNI (*p *< 0.001). A DNI value > 6.5% was a better indicator of severe sepsis/septic shock than C-reactive protein, lactate, white blood cell count, and absolute neutrophil count (sensitivity, 81.3%; specificity, 91.0%; positive predictive value, 88.6%; and negative predictive value, 84.7%). In 36 (82%) of the 44 patients with severe sepsis/septic shock, DNI values were already elevated up to 12 hours before the onset of organ/circulatory failure.

**Conclusions:**

DNI may be used as a marker of disease severity in critically ill patients with sepsis. High levels of DNI may help to identify patients with an impending risk of developing severe sepsis/septic shock.

## Background

Sepsis is the leading cause of death in intensive care units (ICUs) today. In spite of recent advances in antibiotic therapy and general critical care practices, including early goal-directed treatment for septic shock [1], mortality of patients with severe sepsis/septic shock is still substantial [2,3]. Early diagnosis of infection and sepsis before it progresses to organ dysfunction or circulatory failure has crucial impact on the clinical course and outcome of critically ill patients [4]. However, because sepsis is not a final diagnosis, but a clinical syndrome encompassing many heterogeneous conditions with regard to etiology, infection focus, and even presence of infection, there is no gold standard for the detection of sepsis [5].

Many investigators have endeavored to find reliable biomarkers which are useful for the diagnosis and management of sepsis. Ideally, the biomarker should reflect not only the presence of sepsis, but also its severity. Although several biomarkers have been investigated to diagnose infection or sepsis [6,7], no single biologic marker has been shown to reliably identify patients who are at risk of developing severe sepsis or septic shock [8], and tests for these markers are often not widely available at the bedside.

During stress or infection, less mature neutrophil forms enter circulation, including an increased number of bands. This is referred to as a *left-shift*, which is defined as an elevated immature/total granulocyte ratio or an elevated neutrophil band count [9]. The presence of a granulocytic left shift or the enumeration of band neutrophils is still used as a marker of infection or sepsis in clinical practice. Previous studies have demonstrated the clinical usefulness of immature granulocytes or the changes in leukocytes for predicting infection. Granulocyte precursors less mature than bands were reported to be a better predictor of infection than the band counts [10]. Seebach et al. demonstrated a high sensitivity (80%) of morphologic changes in neutrophils, including toxic granulation, Döhle bodies, and cytoplasmic vacuoles, in predicting infection [11]. In a study by Selig et al., myeloid progenitor cells were significantly higher in infectious conditions [12]. Immature granulocyte counts have also been reported as an indicator of sepsis [13,14]. Therefore, the proportion of immature granulocytes may be a better indicator of sepsis than WBC, ANC, or even band neutrophils. However, these granulocyte parameters are difficult to measure accurately and their diagnostic value remains controversial [9]. Consequently, a more reliable and reproducible method to measure immature granulocytes might be useful.

Recent technological advances have led to specific modern automated cell analyzers that can provide information on leukocyte differentials based on cytochemical myeloperoxidase (MPO) reaction and nuclear lobularity of the white blood cells [15-17]. Delta neutrophil index (DNI), the difference between the leukocyte differentials measured in the MPO channel and those assayed in the nuclear lobularity channel, reflects the fraction of circulating immature granulocytes. DNI has been reported to be significantly associated with disseminated intravascular coagulation (DIC) scores, positive blood culture rate, and mortality in patients with suspected sepsis [18]. The data, however, are limited and little is known about the clinical usefulness of DNI in evaluating sepsis severity and in assessing risk of severe sepsis/septic shock in the ICU setting.

In the present study, we investigated DNI values in medical ICU patients with sepsis and evaluated the clinical utility of DNI as an indicator of sepsis severity and as a prediction marker of severe sepsis/septic shock.

## Methods

### Study population

This study was performed in the 30-bed medical ICU of Severance Hospital in Seoul, Korea. The protocol was approved by the Institutional Review Board, and written informed consent was obtained from the patients or next of kin.

Over a period of 6 months (from July 2010 to December 2010), all consecutive patients with clinically diagnosed sepsis at the time of ICU admission were included. Systemic inflammatory response syndrome (SIRS) was defined as two or more of the following conditions: (a) body temperature > 38°C or < 36°C; (b) leukocytosis (> 10,000/μl), leukopenia (< 4,000/μl), or > 10% bands; (c) heart rate > 90 beats/min; and (d) respiratory rate > 24 breaths/min. Sepsis was defined as SIRS with proven or suspected microbial etiology. Severe sepsis was defined as sepsis plus sepsis-induced organ dysfunction or tissue hypoperfusion. Septic shock was defined as an acute circulatory failure characterized by persistent arterial hypotension (systolic arterial pressure below 90 mmHg, mean arterial pressure < 60 mmHg, or a reduction in systolic pressure of > 40 mmHg from baseline despite adequate fluid resuscitation in the absence of other causes of hypotension) [19]. Exclusion criteria were age less than 18 years, pregnancy, patients with hematologic abnormalities, and those who received granulocyte colony stimulating factors, glucocorticoid, or other immunosuppressants before study enrollment.

Patients originated either from the emergency room or from the general wards. At the time of notification for ICU admission of each patient, every effort was made to identify patients with suspected sepsis by dedicated research fellows and attending physicians. Microbiological tests were performed on blood samples, sputum (by nasopharyngeal swab or endotracheal suction), urine specimens, removed catheters, and secretions from other body regions which were suspected to be the infection source. All patients underwent chest radiography and/or high resolution computed tomography scan, magnetic resonance imaging scan, or endoscopy when indicated by attending physicians without interference by the study investigators. On the basis of laboratory, bacteriological, and radiographic findings, "confirmed infection" was determined by a definite source of infection (microbiology confirmed and/or positive culture at a likely focus), and "probable infection" was determined by positive imaging findings, such as an infiltration, cavity, or abscess confirmed by specialized radiologists. Thus, clinically diagnosed sepsis was defined by either confirmed infection or probable infection under the presence of SIRS. During ICU stay, all patients were treated following the international guidelines for management of severe sepsis and septic shock [20].

### Data collection

Baseline demographic data and clinical variables, including age, sex, blood pressure, pulse rate, primary site of infection, blood culture results, presence of severe sepsis/septic shock or overt DIC, average amount of norepinephrine infusion, urine output during the first 24 hours after ICU admission, and hospital mortality were recorded. The presence or absence of mechanical ventilation and renal replacement therapy were also recorded. Simplified Acute Physiology Score (SAPS) 3 [21,22] and Sequential Organ Failure Assessment (SOFA) score [23] were calculated on ICU admission to measure the severity of patient condition. Overt DIC was defined as DIC score ≥ 5 based on the diagnostic criteria by the International Society of Thrombosis and Hemostasis [24].

### DNI and other blood sample measurements

Blood samples for the analyses of DNI and other laboratory parameters were obtained from indwelling arterial catheters or by venipuncture within the first 24 hours of ICU admission. The blood samples were drawn from each patient into EDTA tube, and were immediately transported at room temperature to the chemical laboratory department, and the assay was performed within 1 hour of blood sampling.

A specific type of automatic cell analyzer (ADVIA 2120 Hematology System, Siemens Healthcare Diagnostics, Forchheim, Germany) was used for calculating DNI. This is a flow cytometry-based hematologic analyzer which has two independent white blood cell (WBC) analysis methods, an MPO channel and lobularity/nuclear density channel. First, after lysis of red blood cells (RBCs), the tungsten-halogen based optical system of the MPO channel measures cell size by forward light scatter, and stain intensity by absorbance, thereby counting and differentiating granulocytes, lymphocytes, and monocytes based on their size and MPO content. Second, the laser diode-based optical system of the lobularity/nuclear density channel counts and classifies cells according to size, lobularity, and nuclear density [17,18]. The formula for calculating DNI is as follows: *DNI = [the neutrophil subfraction and the eosinophil subfraction measured in the MPO channel by cytochemical MPO reaction] - [the PMN subfraction measured in the nuclear lobularity channel by the reflected light beam]*. The correlation between DNI values and immature granulocytes by manual counting was reported in a previous study [18]. The measurement of immature granulocytes included promyelocytes, myelocyte, and metamyelocytes, but not blasts.

Complete blood cell counts, including WBC count and absolute neutrophil count (ANC), were measured with an automated analyzer (ADVIA 2120 Hematology System). Prothrombin time (PT), activated partial thromboplastin time (aPTT), D-dimer, and fibrinogen levels were assayed using an STA analyzer (Diagnostica Stago, Asnieres-Sur-Seine, France). Antithrombin III activity was determined using an ELISA kit (Diagnostica Stago). Plasma C-reactive protein (CRP) concentration was measured by direct immunoturbidimetry (CA400, Beckman Coulter, CA, USA). Lactate levels were measured in arterial blood using point-of-care blood gas analyzers (Critical Care Xpress, NOVA biomedical, MA, USA). All measurements were performed according to the manufacturers' instructions.

### Statistical analysis

Continuous variables are presented as the mean ± standard deviation (SD), or when the assumption of normality was violated, as median values and interquartile range. Categorical variables were expressed as absolute and relative frequencies. Comparisons between groups were performed with chi-squared tests for categorical variables and Mann-Whitney U test or Kruskal-Wallis test for continuous variables, as appropriate. We classified patients according to the severity of sepsis (sepsis, severe sepsis, and septic shock), and compared the values of DNI and other laboratory biomarkers among the groups. If statistically significant, *post-hoc *analysis was performed using the Dunn procedure. For comparison, we presented the value of DNI in healthy subjects as controls. The correlation between DNI and other laboratory variables or clinical severity scores was tested by Spearman's method. The effect of increasing quartiles of DNI on the proportion of overt DIC or severe sepsis/septic shock was evaluated by the Cochrane-Armitage trend test. Receiver-operating characteristics (ROC) curves were constructed and the Youden Index method was used to determine the optimal cut-off values for DNI, WBC, ANC, lactate, and CRP for predicting severe sepsis/septic shock. The areas under the curves (AUCs) were calculated to compare the diagnostic performance of each marker. A *p*-value of less than 0.05 was considered statistically significant. Statistical analyses were performed using SAS software, version 9.2 (SAS Institute Incorporated, Cary, NC, USA).

## Results

### Baseline clinical characteristics

A total of 103 patients admitted to ICU were enrolled. Baseline clinical characteristics of the study participants at enrollment are shown in Table 1. Forty-four (42.7%) of 103 patients were diagnosed with severe sepsis/septic shock. Overt DIC occurred in 40 (38.8%) of 103 patients and microorganisms were isolated in 50 (48.5%) of 103 patients. Severe sepsis/septic shock, overt DIC, use of renal replacement therapy, and higher SAPS 3 or SOFA scores were more frequent in nonsurvivors compared to survivors (*p *< 0.05).

**Table 1:** Baseline clinical characteristics of patients by survival

Variables	Survivors (n = 56)	Nonsurvivors (n = 47)	*p*
Age	62.6 ± 17.5	69.4 ± 12.4	0.081
Gender (male)	35 (62.5%)	33 (70.2%)	0.410
Primary site of infection			
Lung	26 (46.4%)	30 (63.8%)	
Intra-abdomen	13 (23.2%)	11 (23.4%)	
Genitourinary	13 (23.2%)	6 (12.8%)	
Skin and soft tissue	3 (5.4%)	0 (0%)	
Others	1 (1.8%)	0 (0%)	
Severe sepsis/septic shock	17 (30.4%)	27 (57.4%)	0.005
Overt DIC	12 (21.4%)	28 (59.6%)	< 0.001
Positive blood culture	24 (42.8%)	26 (55.3%)	0.305
Gram positive	6 (10.7%)	4 (8.5%)	
Gram negative	10 (17.8%)	18 (38.3%)	
Fungus	8 (14.3%)	4 (8.5%)	
None detected	32 (57.2%)	21 (44.7%)	
SAPS 3	62.8 ± 13.6	76.5 ± 14.8	< 0.001
SOFA score	7.9 ± 4.2	11.7 ± 4.8	< 0.001
Mechanical ventilation	31 (55.4%)	29 (61.7%)	0.564
Renal replacement therapy	12 (21.4%)	24 (51.1%)	0.002
Norepinephrine infusion	1.4 ± 3.1	0.7 ± 1.3	0.107
Urine output (24 hours)	2640.7 ± 1461.6	2543.9 ± 1434.6	0.791

Continuous variables are presented as the mean ± standard deviation. Categorical variables are expressed as absolute and relative frequencies within each column.

DIC, disseminated intravascular coagulation; SAPS, Simplified Acute Physiology Score; SOFA, Sequential Organ Failure Assessment; Norepinephrine infusion, average amount of norepinephrine infusion per hour during the first 24 hours in the intensive care unit (ICU); Urine output, total amount of urine output during the first 24 hours in the ICU.

### DNI and other laboratory markers in different subgroups of patients

DNI values were significantly higher in patients with severe sepsis/septic shock (16.1 [7.7-34.2]% vs. 2.3 [0.2-3.9]%; *p *< 0.001) and overt DIC (10.8 [4.7-17.4]% vs. 2.6 [0.8-6.7]%; *p *< 0.001) than in patients without severe sepsis/septic shock and overt DIC, respectively. Plasma lactate levels also showed similar pattern between the groups. In contrast, there was considerable overlap in WBC, ANC, and CRP levels between the groups (Table 2).

**Table 2:** Delta neutrophil index and other laboratory markers in different subgroups of patients

Variables	Without severe sepsis/septic shock (n = 59)	Severe sepsis/septic shock (n = 44)	
WBC, 10^3^/uL	11610 (8940-19210)	13820 (8870-21505)	0.655
ANC, 10^3^/uL	10100 (6230-14440)	11805 (7103-19238)	0.394
DNI, %	2.3 (0.2-3.9)	16.1 (7.7-34.2)	< 0.001
Lactate, mmol/L	1.8 (1.2-3.65)	4.0 (2.1-9.9)	< 0.001
CRP, mg/dL	11.3 (4.6-19.2)	16.2 (9.8-28.3)	0.017

	Without overt DIC (n = 63)	Overt DIC (n = 40)	

WBC, 10^3^/uL	12780 (9230-20180)	11805 (8150-22320)	0.768
ANC, 10^3^/uL	10780 (6870-16890)	9765 (4775-17438)	0.629
DNI, %	2.6 (0.8-6.7)	10.8 (4.7-17.4)	< 0.001
Lactate, mmol/L	1.9 (1.2-3.5)	4.7 (2.4-14.2)	< 0.001
CRP, mg/dL	13.6 (5.4-24.3)	15.1 (2.8-23.3)	0.854

Data are expressed as median (interquartile range).

WBC, white blood cell count; ANC, absolute neutrophil count; DNI, delta neutrophil index; CRP, C-reactive protein

### DNI and other markers in SIRS, sepsis, and severe sepsis/septic shock group

**Figure 1:**
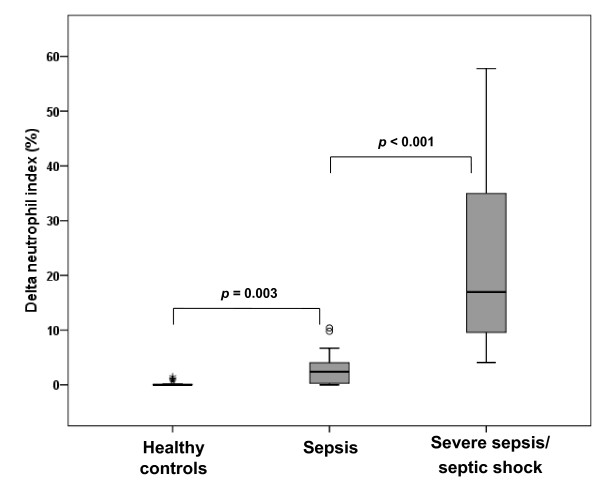
**Delta neutrophil index (DNI) values in subgroups with different sepsis severity**. Box plots represent the 25^th ^and 75^th ^percentiles, with the internal horizontal lines showing the median. DNI values increased with disease severity from the control group to the severe sepsis/septic shock group.

When patients were classified into subgroups according to the severity of sepsis (control group: n = 30, sepsis group: n = 59, severe sepsis/septic shock group: n = 44), DNI values increased according to disease severity from the control group to severe sepsis/septic shock group. In detail, median values and interquartile range of DNI were 0 (0-0.1)% in the healthy control group, 2.8 (0.5-5.3)% in the sepsis group, and 16.9 (9.5-35.6)% in the severe sepsis/septic shock group (Figure 1). Similar trend was also shown in lactate levels (median values of 0.9, 1.4, and 4.1 mmol/L). In contrast, significant incremental trend was not observed in WBC (median values of 7360, 13610, and 12970/mm^3^), ANC (median values of 4515, 9720, and 11665/mm^3^), or CRP (median values of 1.4, 16.1, and 15.7 mg/dL).

### Relationship between DNI and laboratory variables/clinical severity scores

**Figure 2:**
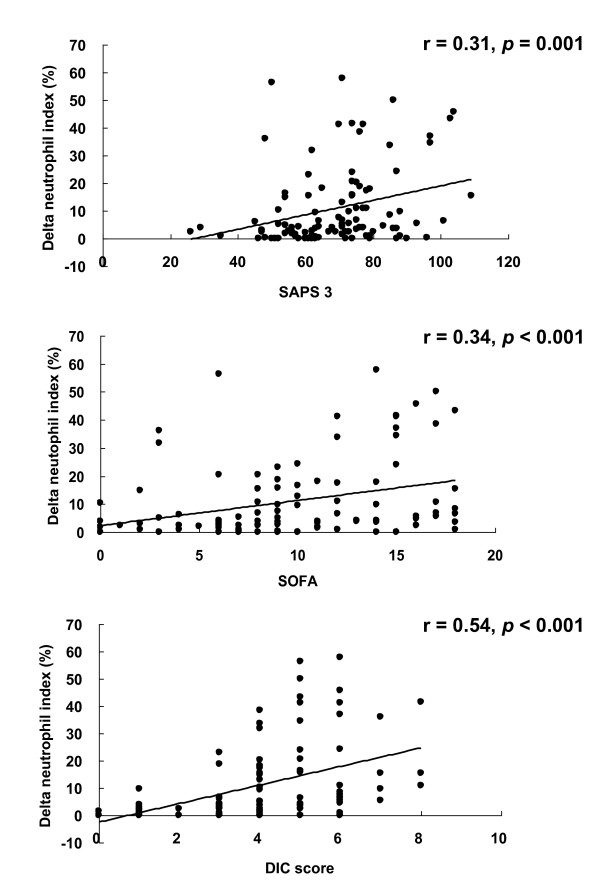
**Correlation of delta neutrophil index with clinical severity scores**. SAPS, Simplified Acute Physiology Score; SOFA, Sequential Organ Failure Assessment; DIC, disseminated intravascular coagulation.

DNI correlated positively with clinical severity scores; SAPS 3 score (r = 0.31, *p *= 0.001), SOFA score (r = 0.34, *p *< 0.001), and DIC score (r = 0.54, *p *< 0.001; Figure 2). DNI did not correlate with WBC count (r = 0.07, p = 0.467) or ANC (r = 0.13, *p *= 0.184).

### Proportion of overt DIC and severe sepsis/septic shock according to the quartiles of DNI values

**Figure 3:**
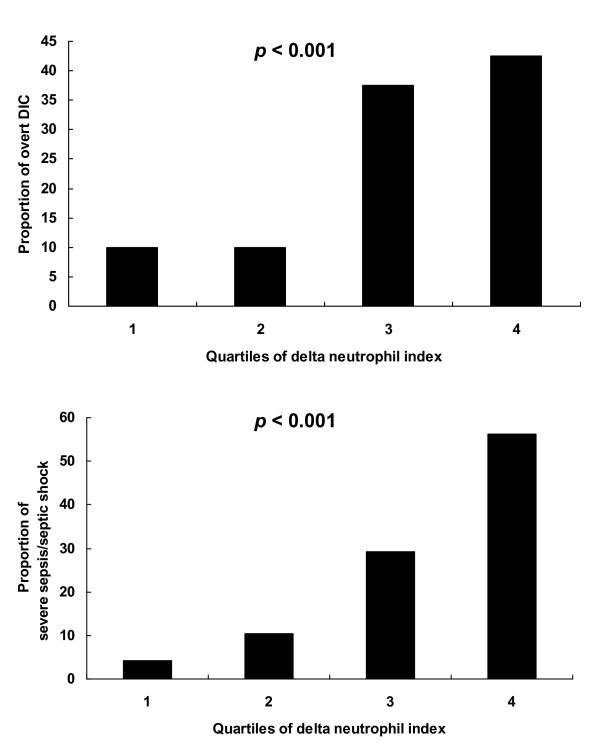
**Proportion of overt disseminated intravascular coagulation (DIC) and severe sepsis/septic shock according to the quartiles of delta neutrophil index (DNI) values**. The first, second, and third quartile values of DNI values were 1.6, 4.3, and 15.5%, respectively. The proportion of patients with overt DIC and severe sepsis/septic shock increased in association with increasing quartiles of DNI values (*p *< 0.001). *p*-values were derived from the Cochrane-Armitage trend test.

As shown in Figure 3, a monotonic increase in the proportion of patients with overt DIC and severe sepsis/septic shock was observed in association with increasing quartiles of DNI values (*p *< 0.001). The first, second, and third quartile values of DNI values were 1.6, 4.3, and 15.5%, respectively.

### Performance of DNI and other laboratory markers in identifying severe sepsis/septic shock

**Figure 4:**
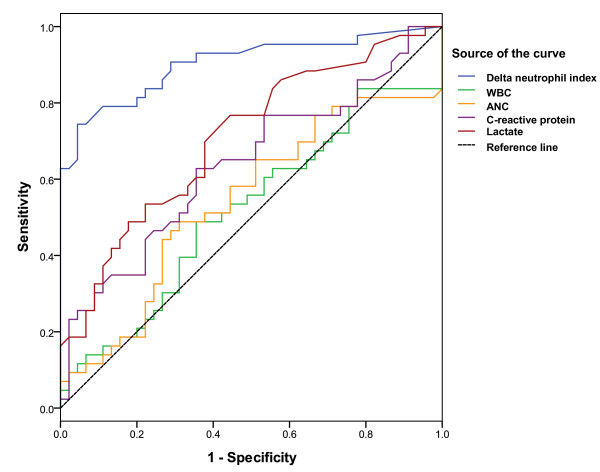
**Receiver operating characteristics (ROC) curves of delta neutrophil index (DNI) and other laboratory markers for differentiating between the presence and absence of severe sepsis/septic shock**. Areas under ROC were 0.92 (95% CI, 0.86-0.98) for DNI, 0.53 (95% CI, 0.41-0.64) for white blood cell (WBC), 0.76 (95% CI, 0.61-0.82) for lactate, and 0.64 (95% CI, 0.53-0.75) for C-reactive protein (CRP). The accuracy of DNI for discriminating between the presence and absence of severe sepsis/septic shock was superior to those of other laboratory markers (*p *< 0.05).

A cut-off value of 6.5% for DNI exceeded those for any other laboratory markers for differentiating the presence and absence of severe sepsis/septic shock (Table 3). As shown in Figure 4, ROC curves demonstrated that DNI was the best indicator of severe sepsis/septic shock, with an AUC of 0.92 (95% confidence interval [CI], 0.86-0.98). The accuracy of DNI for differentiating between the presence and absence of severe sepsis/septic shock was higher than those of other laboratory markers (*p *< 0.001 for DNI vs. WBC; *p *= 0.002 for DNI vs. ANC; *p *= 0.02 for DNI vs. lactate; and *p *= 0.009 for DNI vs. CRP).

**Table 3:** Performance of delta neutrophil index and other laboratory markers in differentiating between the presence and absence of severe sepsis/septic shock

Variables	Cut-off level	Sensitivity (%)	Specificity (%)	Positive predictive value (%)	Negative predictive value (%)
DNI	6.5%	81.3%	91%	88.6%	84.7%
WBC	16590/mm^3^	45.8%	69.1%	56.4%	59.4%
ANC	14100/mm^3^	45.8%	72.7%	59.4%	60.6%
Lactate	2.1 mmol/L	76.7%	57.1%	61.1%	73.7%
CRP	15.4 mg/dL	59.6%	66%	62.2%	63.5%

DNI, delta neutrophil index; WBC, white blood cell count; ANC, absolute neutrophil count; CRP, C-reactive protein.

### Time course of DNI values before and after the onset of severe sepsis/septic shock

**Figure 5:**
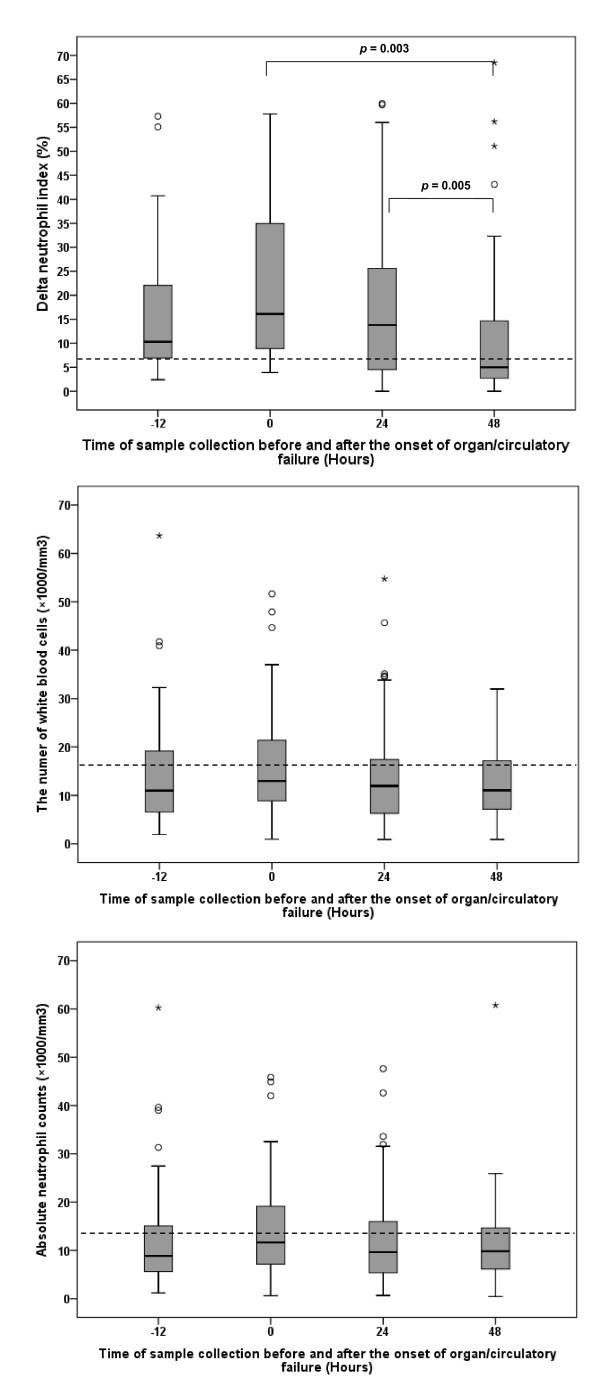
**Time course of delta neutrophil index (DNI) values, white blood cell (WBC) counts, and absolute neutrophil counts (ANC) before and after the onset of severe sepsis/septic shock**. On the basis of the optimal cut-off values (DNI, 6.5%; WBC, 16590/mm^3^; and ANC, 14100/mm^3^) presented in Table 3, DNI values already elevated before the onset of organ/circulatory failure in 36 (82%) of the 44 patients with severe sepsis/septic shock, and decreased over time after ICU admission (*p *< 0.001). In contrast, there were no changes in WBC counts (*p *=0.235) or ANC (*p *= 0.223) over time.

Although we enrolled patients in a consecutive manner, we could also check the DNI values of them up to 12 hours before the onset of organ dysfunction or significant hypotension (retrospective data collection before ICU admission). Using the cut-off levels in Table 3 DNI values already increased before the onset of organ/circulatory failure in 36 (82%) of the 44 patients with severe sepsis/septic shock, and decreased over time after ICU admission (*p *< 0.001). This evolutionary pattern was not observed in WBC (*p *=0.235) or ANC (*p *= 0.223; Figure 5).

## Discussion

In this study, we demonstrated that DNI, which reflects the number of circulating granulocyte precursors in the blood, correlated with the severity of sepsis in critically ill patients admitted to the medical ICU. The elevation of DNI value preceded the onset of organ/circulatory failure, thus contributing to identifying patients with an impending risk of developing severe sepsis/septic shock.

In the present study, increased DNI values at the time of ICU admission were significantly associated with the presence of severe sepsis/septic shock and overt DIC. These results are consistent with a previous report by Nahm et al. who concluded that DNI was closely related to the presence of overt DIC, bacterial isolation rate, and mortality in patients with suspected sepsis [18]. In another study by Ansari-Lari et al., the percentage of immature granulocytes correlated better with infection and positive blood culture results than the WBC count [13], but the sensitivity was low (40% sensitivity at 90% specificity). Consequently, the authors suggested that high cut-off levels for the percentage of immature granulocytes might be required to reliably predict infection or positive blood culture results. In that study, however, the authors tested the usefulness of immature granulocytes as a predictor of infection, not sepsis. In contrast, we evaluated the clinical usefulness of DNI in sepsis (including severe sepsis/septic shock). These different inclusion criteria may have yielded different sensitivity, specificity, and even higher optimal cut-off value for DNI in our results.

Severe forms of sepsis are associated with DIC, and DIC is often present before the onset of sepsis [25]. We found that DNI correlated with SAPS 3, SOFA, and DIC score. Similar to our results, a previous report by Nahm et al. also demonstrated a significant relationship between DNI and DIC-related parameters, including platelet count, PT, aPTT, and antithrombin III [18]. These findings suggest that DNI may be linked to a hypercoagulable state which is associated with sepsis, and that DNI may reflect the clinical severity of critically ill patients with sepsis.

In our results, DNI values were higher in the severe sepsis/septic shock group compared to the sepsis group. In line with this finding, the Cochrane-Armitage trend test also showed that the proportion of patients with overt DIC and severe sepsis/septic shock gradually increased with the increase in DNI values. Importantly, the proportion abruptly increased when DNI was higher than 4.3% (the second quartile value of DNI). In our ROC analysis, the diagnostic value of DNI for severe sepsis/septic shock was superior to WBC, ANC, or other widely available laboratory markers. The optimal cut-off value of DNI for predicting severe sepsis/septic shock was 6.5%. Taken together, our data suggest that careful attention may be required in patients with suspected infection for possible concomitant DIC and/or severe sepsis/septic shock if DNI value increases up to 4-6% or more.

A recent study showed that mortality was correlated to the duration of hypotension before the start of antibiotic treatment [4]. Therefore, it is very important role for clinicians to identify patients who are at risk of developing severe sepsis/septic shock before the signs of organ dysfunction or circulatory failure appear. In the present study, DNI values had already increased before the onset of organ/circulatory failure in 82% of the patients with severe sepsis/septic shock, suggesting that DNI may help to identify patients with an imminent risk of developing severe sepsis/septic shock. Given that the process of granular leukocyte differentiation starts from immature granulocyte formation, the change in DNI may have preceded the change in absolute numbers of WBC or neutrophil, thus contributing to predicting the development of severe sepsis/septic shock. Based on our data, we suggest that the finding of an increased DNI value should alert clinicians to start fluid resuscitation or to change antibiotic treatment.

Several limitations of our study should be mentioned. First, this was a single-center study and the sample size was relatively small. Second, the elevation of immature granulocytes is not specific for infection and may be observed in various other conditions, including myeloproliferative disorders, chronic inflammatory diseases, tissue damage, acute hemorrhage, and neoplasia [9]. Because DNI is also one of the leukocyte-related parameters, there may be a lack of sensitivity or specificity for DNI as a severity marker of sepsis in this group. Third, we did not evaluate the comparative advantage of using DNI over procalcitonin which may have a role in reducing antibiotic exposure of critically ill patients [26] and serve as a useful complementary comparator for prediction of survival outcome in postoperative patients with severe sepsis [27]. Because the main focus of the present study was DNI, and the decision to check procalcitonin level was left to the attending physicians, the data for procalcitonin were obtained only from 63 (61%) of 103 patients, thereby yielding relatively lower discriminative power (AUC 0.65 [95% CI, 0.54-0.77], data not shown) compared to the previous reports [28]. More studies with large number of patients are required to validate the clinical usefulness of DNI as a severity and prediction marker of sepsis. Further studies are also warranted to investigate the additional benefit of combining DNI with other biomarkers, such as procalcitonin, to improve their predictive power.

## Conclusion

The present study demonstrated that DNI, which reflects the proportion of immature granulocytes in circulating blood, correlates with disease severity of sepsis in critically ill patients admitted to the medical ICU. For assessing the risk of severe sepsis/septic shock, DNI may be a better predictive marker than other leukocyte-derived parameters. The elevation of DNI value may help to identify patients with possible co-existent DIC and patients with an imminent risk of developing severe sepsis/septic shock. Thus, incorporating the immature granulocyte assay into the routine algorithms may improve the early detection of severe sepsis/septic shock.

## List of abbreviations

DNI: delta neutrophil index; DIC: disseminated intravascular coagulation; ICU: intensive care unit; MPO: myeloperoxidase; SIRS: systemic inflammatory response syndrome; SAPS: Simplified Acute Physiology Score; SOFA: Sequential Organ Failure Assessment; WBC: white blood cell; RBC: red blood cell; PT: prothrombin time; aPTT: activated partial thromboplastin time; CRP: C-reactive protein; ROC: Receiver-operating characteristics; AUC: areas under the curves.

## Competing interests

The authors declare that they have no competing interests.

## Authors' contributions

BH Park carried out screening and statistical analysis of the data and participated in the writing of the manuscript. WJ Jung, SH Lee, SK Lee, and SY Kim carried out screening and acquisition of data. JY Jung participated in the acquisition of data and statistical analysis. YA Kang, MS Park participated in the study design and the analysis and interpretation of data. SK Kim and J Chang participated in the study design, analysis and interpretation of data and critical revision of the manuscript for important intellectual content. YS Kim participated in the study design, analysis and interpretation of data and the writing of the manuscript. All authors read and approved the final manuscript.

## Pre-publication history

The pre-publication history for this paper can be accessed here:

http://www.biomedcentral.com/1471-2334/11/299/prepub
